# INTERGENERATIONAL DIFFERENCES: HPV KNOWLEDGE AND PERCEIVED HEALTH STATUS AT A MIDWESTERN UNIVERSITY, 2016–2022

**DOI:** 10.1093/geroni/igae098.3410

**Published:** 2024-12-31

**Authors:** Whitney Nesser, Scott Snyder

**Affiliations:** Indiana State University, Terre Haute, Indiana, United States; University of Alabama at Birmingham, Birmingham, Alabama, United States

## Abstract

Studies of Human Papillomavirus (HPV) knowledge and health status have been conducted with university students, but typically exclude faculty and staff. The study aim was to assess HPV knowledge and perceived health status among students, staff, and faculty at a Midwestern university. Three cross-sectional surveys were conducted at a Midwestern university in 2016, 2019, and 2022 assessing HPV knowledge and perceived health status. A total of 2678 student, staff, and faculty responses were received. Perceived health status was measured using a standard health status question. Knowledge of HPV was measured as total score on a 15-item true/false test. There were 145 respondents 61 years or older. A factorial ANOVA revealed that HPV knowledge was significant for age group (p <.001) but not cohort or interaction. Respondents 61 and older performed better than those aged 18-21 but lower than groups between ages 27-45. Main effects for minority status and age group (p <.001) were found but no interaction (p =.655). No cohort effect was found for perceived health status. Chi-square analyses showed perceived health status relates to age group (p <.01) with significantly more individuals 61 and older rating their health status as excellent than expected (SD = 2.6). Perceived health status for this age group was independent of cohort (p =.22). These findings highlight the need to increase HPV education to individuals under age 27, further investigate health status perceptions in individuals 61 and older, and explore perceived health status in minority respondents.

